# Histopathological evaluation of synovial tissue in advanced osteoarthritis: A retrospective study based on total knee arthroplasty specimens

**DOI:** 10.1097/MD.0000000000044152

**Published:** 2025-08-29

**Authors:** Ilkay Cinar

**Affiliations:** a Department of Pathology, Faculty of Medicine, Giresun University, Merkez-Giresun, Türkiye.

**Keywords:** fibrosis, inflammation, knee, osteoarthritis, synovitis

## Abstract

Osteoarthritis (OA) is a chronic disease characterized by pain, swelling, and joint stiffness, affecting all components of the joint and periarticular tissues. This study aimed to evaluate the histomorphological features of synovial tissue obtained during total knee arthroplasty in patients with grade 4 primary OA and to explore the relationship between synovitis scores and associated pathological changes. This retrospective, single-center cohort study included 115 patients who underwent total knee arthroplasty for grade 4 primary OA between 2020 and 2023. Histological evaluations were performed on archived hematoxylin and eosin stained sections. Synovitis scores were calculated using a semiquantitative scoring system that assessed synovial lining hyperplasia, inflammatory infiltrate, and stromal fibrosis. Additional features such as vascularity, edema, multinucleated giant cells, and calcification were also evaluated. Statistical analyses included descriptive statistics, Chi-squared tests, and Spearman correlation. Synovial inflammation was classified as mild in 73 patients (63.5%), moderate in 37 (32.2%), and severe in 5 (4.3%). Fibrosis was the most prominent histological finding, with severe fibrosis observed in 67 patients (58.3%) and moderate fibrosis in 40 patients (34.8%). The mean synovitis score was 4.9. While inflammation was generally mild to moderate, fibrosis and stromal proliferation contributed significantly to the overall score. A significant correlation was found between synovitis score and age (*P* = .039; ρ = 0.193), as well as with the presence of multinucleated giant cells (*P* < .001). Synovial fibrosis appears to be a defining feature of advanced-stage OA and may significantly contribute to pain. However, synovial inflammation should also be recognized as a key component in the disease’s pathophysiology. Standardized histopathological scoring systems improve the consistency of synovial assessment and can guide future research. Longitudinal studies investigating the progression of both fibrosis and inflammation across OA stages may support the development of targeted therapies.

## 1. Introduction

Osteoarthritis (OA) is a chronic disease characterized by pain, swelling, and stiffness in the joints and affecting all structures of the joint and periarticular tissues. It is a major cause of joint pain and disability.^[1]^ The primary sites affected are the synovial joints, including the knees, hips, and hands, with the knee being the most commonly affected joint.^[2]^ It is estimated that one-third of people over the age of 65 suffer from this disease. In addition to reducing quality of life, OA poses a significant challenge on healthcare systems worldwide.^[3,4]^

The diagnosis of OA is generally based on radiographic rather than clinical features. Radiographic criteria were proposed by Kellgren and Lawrence in 1957 and were later adopted by the World Health Organization at a symposium held in Milan in 1961.^[5]^ Previous studies have primarily focused on changes in the continuity of articular cartilage and chondrocyte pathology. However, OA causes pathological changes not only in the cartilage but also in the synovium, bone, ligaments, muscles, and fibrocartilage structures.^[6]^

Since adult articular cartilage is avascular and aneural hyaline cartilage, pathological changes in non-cartilaginous structures – such as the joint capsule, synovium, subchondral bone, ligaments, and periarticular muscles – emerge as the primary sources of pain in OA.^[7,8]^

The term synovium refers to the soft tissue that lines the inner surface of synovial (diarthrodial) joints, tendon sheaths, and bursae. This tissue consists of a continuous layer of cells known as the intima, and an underlying tissue layer called the subintima.

The intima is primarily composed of macrophages and fibroblasts. The subintima contains additional fibroblasts, macrophages, adipocytes, and blood vessel.^[9,10]^

The infrapatellar fat pad (IFP) of the knee joint is located between the patella and the tibia, with its posterior surface – facing the joint – covered by synovium. In recent years, its role in OA has become a subject of growing research interest. It has been demonstrated that the IFP and the synovial membrane function as an anatomo-functional unit and that the IFP serves as a source of adipocytokines, which may contribute to inflammation in OA.^[11]^ The IFP surrounds other structures within the knee and is highly vascularized and innervated. Therefore, inflamed or injured it may become a significant source of pain. The synovial membrane is associated with intra-articular inflammation and cellular changes, both of which contribute to pain. Inflammation of these tissues involves numerous molecules that act as inflammatory and neuropathic mediators.^[12]^ Cells within the IFP and the synovial membrane produce cytokines such as IL-6, IL-8, and TNF-α. These cytokines trigger inflammation in the surrounding tissues and contribute to pain, inflammation, and tissue degeneration. Elevated IL-6 levels and increased vascularization promote the progression of inflammation and lead to structural changes.^[13]^

Today, the predominant perspective is that synovial inflammation plays a crucial role in the pathophysiology of OA. Synovial inflammation has been identified as a trigger for OA, leading to an increase in synovial thickness and infiltration of inflammatory cells including macrophages, lymphocytes, and natural killer cells.^[14,15]^

Currently, there are no specific medications available to cure OA. Symptoms are typically managed through a combination of non-pharmacological approaches and nonsteroidal anti-inflammatory drugs. However, the effectiveness of current analgesic treatments is limited, and they are often associated with serious side effects. As a result, joint replacement remains the only viable long-term solution.^[4,16]^

This study examined the histomorphological features of the synovium in patients with grade 4 OA and compared the findings with those reported in the existing literature. OA is a complex and multifactorial disease. Inflammation of the IFP and the synovial membrane may play a central role in the development of pain. Further research in these areas could help identify new therapeutic targets. Such studies may also pave the way for novel strategies in pain management by addressing both peripheral and central mechanisms of pain.

## 2. Methods

### 2.1. Study design and setting

This retrospective, single-center observational study was conducted at the Department of Pathology, Giresun University Faculty of Medicine. It was designed to investigate the histomorphological characteristics of synovial tissue obtained from patients who underwent total knee arthroplasty due to grade 4 primary OA between 2020 and 2023. Ethical approval was obtained from the Giresun University Ethics Committee (approval no: 24.04.2024/02), and the study was carried out in accordance with the Declaration of Helsinki.

### 2.2. Data sources and measurements

Patients who underwent total knee replacement surgery due to grade 4 primary OA were identified through hospital records. Histopathological specimens were retrieved from the pathology department’s block and slide archives. Demographic and clinical data, including age and sex, were extracted from medical records. Radiological and clinical evaluations were based on the diagnostic criteria established by Altman et al, and only patients meeting these criteria were included in the study.^[17]^ Patients with secondary OA due to metabolic disorders, trauma, tumors, or other non-primary causes were excluded.

### 2.3. Histopathological analysis

Archived hematoxylin and eosin (HE) stained preparations were reevaluated. Additional sections were obtained from paraffin blocks and stained with HE and Masson trichrome (MTC) when necessary. Synovial tissue samples were processed using standard histological procedures. Samples were fixed in 10% buffered formaldehyde for 24 to 48 hours, dehydrated, and embedded in paraffin blocks. Sections 5 µm thick were cut using a microtome and mounted on glass slides. HE staining was used to evaluate the general histopathological architecture of the synovium, including the synovial lining and the presence of inflammatory cells. Additionally, MTC staining was performed to assess the extent of fibrosis. The MTC technique enables visualization of collagen fibers, which appear blue, allowing for the quantification of fibrosis.^[18]^

### 2.4. Evaluation methods

Histomorphological evaluations were conducted using a Nikon Eclipse-ci light microscope at various magnifications (×100, ×200, ×400) by an expert pathologist. The following histopathological features were assessed.

In the histological evaluation, a 3-tier scoring system proposed by Krenn et al was utilized, focusing on fundamental synovial changes associated with inflammation.^[19]^ Under the light microscope, 3 key parameters were identified and scored as follows:

1 Enlargement of the synovial lining cell layer:

- 1 point for 2 to 3 cell layers
- 2 points for 4 to 5 cell layers
- 3 points for more than 5 cell layers

2 Stromal fibrosis/cellularity:

- 1 point for a slight increase
- 2 points for a moderate increase with occasional giant cells
- 3 points for a marked increase with pannus formation

3 Degree of inflammatory infiltrate:

- 1 point for sparse perivascular inflammation
- 2 points for perivascular inflammation along with moderate inflammation forming follicular aggregates
- 3 points for intense band-like inflammation

Each parameter was scored independently, and a total synovitis score was calculated by summing the individual parameter scores.

The sum synovitis score was categorized as follows and is depicted in Table 1.

**Table 1:** Schematic of histopathological assessment of chronic synovitis.

A: Enlargement of synovial lining cell layer	B: Stromal fibrosis and cellularity	C: İnflammatory infiltrate
0 point: 1 layer	0 point: normaly	0 point: no inflammation
1 point: 2–3 layer	1 point: the cellularity and fibrosis is slightly increase	1 point: few mostly perivascular inflammation
2 point: 4–5 layer	2 point: moderately increase cellularity, some multinucleated giant cells	2 point: numerous inflammation, some follicle like aggregates
3 point: more than 5 layer	3 point: greatly increased cellularity and giant cells, pannus formation	3 point: dense band like infiltrate

Synovitis scores: A + B + C. sum 0–1: no synovitis, sum 2–4: low grade synovitis, and sum 5–9: high grade synovitis.

Additionally, perivascular stromal edema, papillarity, presence of granulomas, calcification, vascularity, and fibrin accumulation were also assessed using a semiquantitative scoring method like the approach used for synovitis. These features were evaluated at sites showing the most pronounced histopathological alterations, as inflammatory changes often exhibit heterogeneity across different regions of the synovium. By focusing on the most prominent pathological changes, the scoring system aimed to ensure the accurate and consistent grading of synovial inflammation and fibrosis.

Statistical analysis: all statistical analyses were conducted using IBM SPSS Statistics version 23.0 for Windows (IBM Corp., Armonk). Descriptive statistics were used to summarize the data. Continuous variables are reported as medians with interquartile ranges, while categorical variables are presented as frequencies and percentages. Comparisons between proportions were performed using the Chi-squared test and Spearman correlation test. A *P*-value of <.05 was considered statistically significant.

## 3. Results

In total, 115 patients were included in this study. Nineteen patients (16.5%) were men, and 96 (83.5%) were women. The median age was 66 years (range: 50–82), and the mean age was 66 years. The mean BMI was 28.7 ± 12.2 kg/m^2^.

### 3.1. Histopathological changes

Synovial lining cells generally exhibited 2 rows (78%) and 3 rows (31%). The synovial lining score was 1 in 94.8% of the patients.

Synovial inflammation was classified as mild in 73 patients (63.5%), moderate in 37 patients (32.2%), and severe in 5 patients (4.3%).

The most significant histopathological change was an increase in fibrous tissue. Severe fibrosis was detected in 67 patients (58.3%), moderate fibrosis in 40 patients (34.8%), and mild fibrosis in 8 patients (7%).

**Figure 1.**
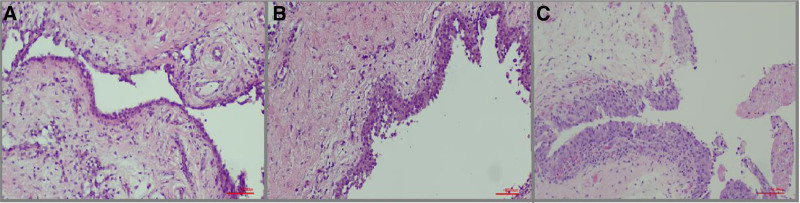
Representative appearances of synovial lining hyperplasia in OA patients. (A) Score 1: synovial lining composed of 2 to 3 cell layers (HE, 200×). (B) Score 2: synovial lining composed of 4 to 5 cell layers (HE, 200×). (C) Score 3: synovial lining with more than 5 cell layers (HE, 400×). HE = hematoxylin and eosin, OA = osteoarthritis.

**Figure 2.**
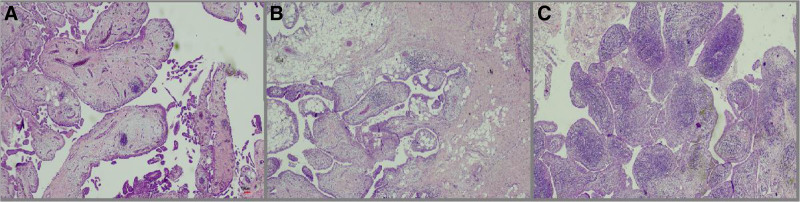
Representative appearances of synovial inflammation in OA patients. (A) Inflammation Score 1: sparse perivascular inflammation (HE, 200×). (B) Inflammation Score 2: moderate infiltration (hematoxylin and eosin, 200×). (C) Inflammation Score 3: dense and diffuse inflammatory cell infiltration (HE, 200×). HE = hematoxylin and eosin, OA = osteoarthritis.

**Figure 3.**
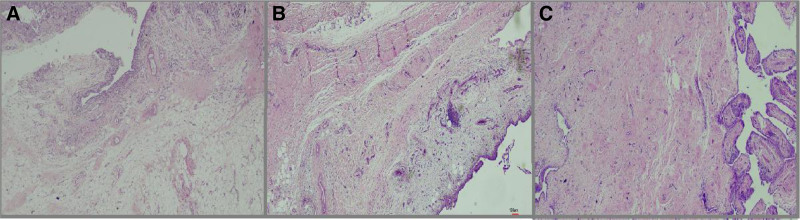
Representative appearances of synovial fibrosis in OA patients. (A) Fibrosis Score 1: Slight increase in fibrous tissue (HE, 200×). (B) Fibrosis Score 2: moderate fibrotic expansion with occasional multinucleated giant cells (HE,200×). (C): Fibrosis Score 3: marked fibrosis with pannus formation (HE, 400×). HE = hematoxylin and eosin, OA = osteoarthritis.

**Figure 4.**
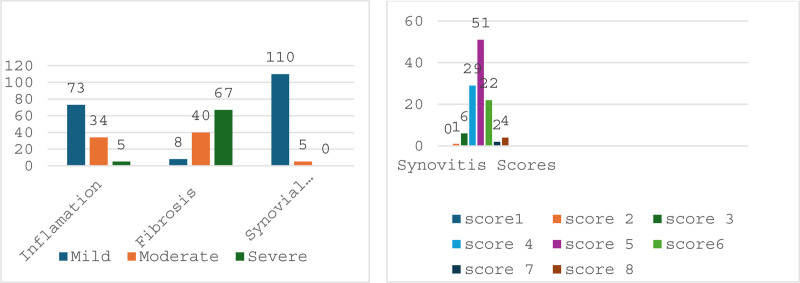
Graphs show the distribution of inflammation, fibrosis, and synovial lining, as well as the total synovitis scores. In advanced-stage OA, inflammation was mostly mild, fibrosis was severe, and the number of synovial cells was 1 to 2. The most common total synovitis score was 5. OA = osteoarthritis.

The synovitis score was calculated as the sum of the synovial lining score, the inflammation severity score, and the fibrosis score. Representative examples of scores 1, 2, and 3 are shown in Figures 1 to 3 respectively. The mean synovitis score was 4.9, while the median score was 5. Additionally, scores of 4 and 6 were also common: 51 patients (44.3%) had a score of 5, 29 patients (25.2%) had a score of 4, and 22 patients (19.1%) had a score of 6 (Fig. 4). Accordingly, a high-grade synovitis score was detected in patients with grade 4 OA, with values close to the border between high-grade and low-grade synovitis. While inflammation was mostly mild or moderate, fibrosis and stromal proliferation were the main contributors to the increase in the synovitis score. A positive correlation was found between synovitis score and age (*P* = .039; ρ = 0.193).

A moderate increase in vascularity was observed in the synovial stroma of 62 patients (40%), whereas a severe increase in vascularity was observed in 45 patients (38.3%). Mild edema was detected in 46 patients (40%), moderate edema in 31 patients (27%), and severe edema in 10 patients (8.7%). No edema was observed in 28 patients (24.3%). Papillary hyperplasia was observed in 107 patients (93%), while it was absent in 8 patients (7%). Mild fibrin accumulation was observed in 17 patients (14%), moderate accumulation in 6 patients (5.2%), and severe fibrin accumulation in 8 patients (7%). No fibrinoid material was observed in 84 patients (73%).

### 3.2. Additional observations

**Figure 5.**
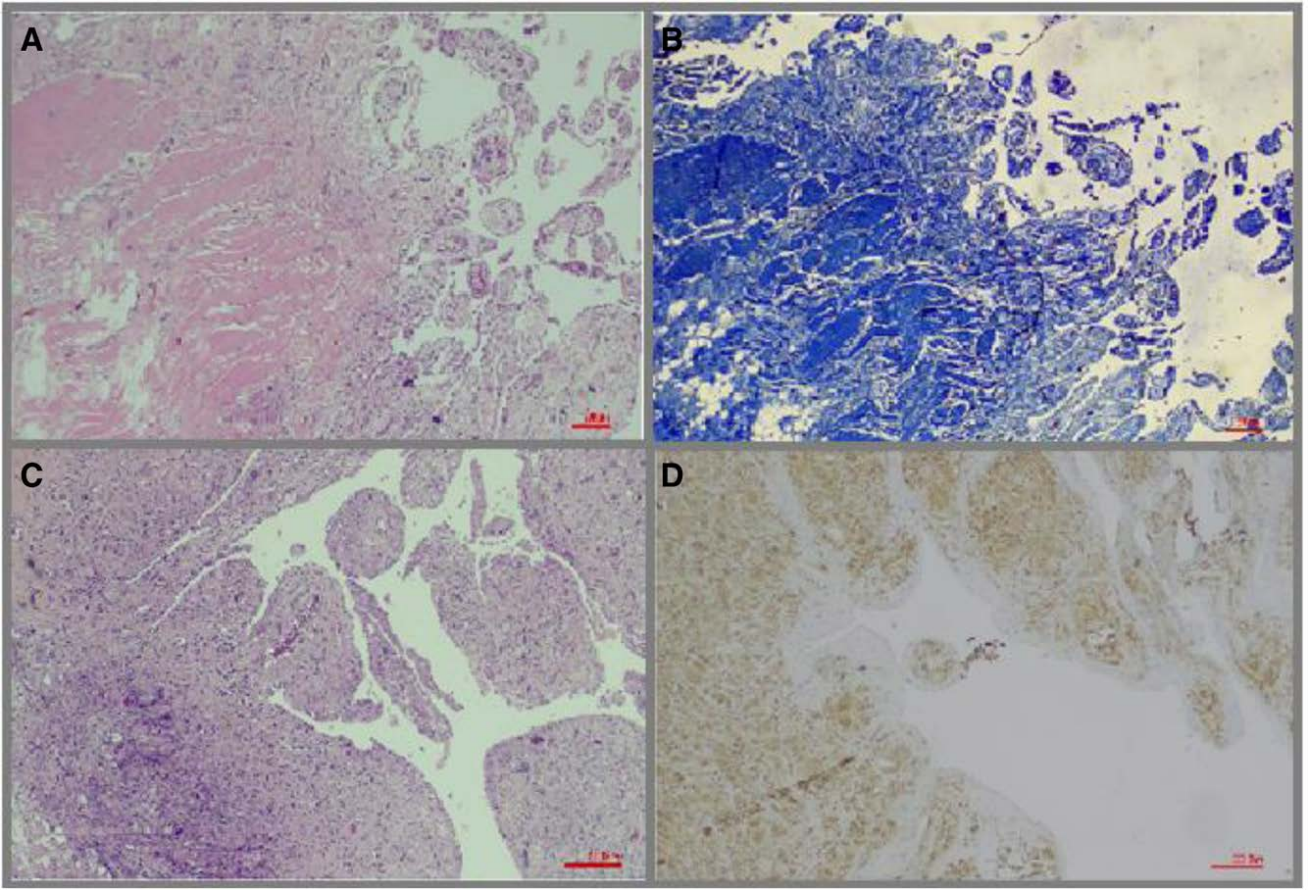
Some histopathological features in advanced-stage OA. (A) Fibrosis was the most prominent finding. Hematoxylin and eosin staining highlights dense fibrotic areas (HE, 400×). (B) Collagen fibers in the synovium are visualized as blue structures with Masson Trichrome staining. The fibrosis score in this case was 3 (MTC,400×). (C) MGC’s were predominantly observed in patients with high synovitis scores (HE, 400×). (D) MGC’s are visualized using CD 68 immunohistochemical staining (CD 68, 400×). HE = hematoxylin and eosin, MGC = multinucleated giant cell, MTC = Masson trichrome, OA = osteoarthritis.

Among patients with high synovitis scores, multinucleated giant cells (MGCs) were observed in the stromal tissue of 14 patients (12.2%) and granulomas were detected in 3 patients (2.6%). When the relationship between histomorphological data was evaluated using the Chi-square test, a significant correlation was found between synovitis score and the presence of MGCs (*P* < .001). Stromal calcification was observed in 15 patients (13%), while cartilage and bone metaplasia were identified in 6 patients (5.2%). The mean age of patients with calcification was 70 years (±7.65), which was older compared to the general study population, and the majority (86%) were female. Fibrosis was more common in this group, with 80% showing a fibrosis score of 3 and 20% showing a score of 2. Synovitis scores in these patients were also high, ranging between 5 and 6. These findings suggest that stromal calcification and bone metaplasia in the synovium may develop in elderly individuals who have been exposed to the disease for a longer duration. In Figure 5, fibrosis is demonstrated using MTC staining, and MGCs are shown using CD68 immunostaining.

The histomorphological findings are summarized in Table 2.

**Table 2:** General histomorphological findings.

	Absent	Mild	Moderate	Severe
Synovial inflammation	0	73 (65.5%)	37 (32.2%)	5 (4.3%)
Fibrosis	0	8 (7%)	40 (34.8%)	67 (58.3)
Vascularity	0	8 (7%)	62 (53.9%)	45 (39.1%)
Edema	28 (24%)	46 (40%)	31 (27%)	10 (8.7)
Papillarity	8 (7%)	17 (14.8%)	46 (40%)	44 (38.3%)
Fibrin accumulation	84 (73%)	17 (14.8%)	6 (5.2%)	8 (7%)

## 4. Discussion

OA is no longer considered merely a degenerative cartilage disease; rather, it is now recognized as a condition that affects the entire joint structure, including the synovium, subchondral bone, ligaments, and the IFP.^[12]^ Recent studies have increasingly emphasized the central role of synovial inflammation and fibrosis in the progression and symptomatology of OA.^[17,20–22]^ There is often a discordance between clinical radiographic findings and patient-reported symptoms in OA, and synovitis has been proposed as a potential explanation for this discrepancy.^[20]^ In this context, recent studies have focused on the synovium and the IFP, consistently reporting the presence of low-grade chronic inflammation, particularly within the synovial membrane.^[17,19,21]^ In synovial inflammation, the role of synovial cellular components – especially activated synovial macrophages and the immune system they stimulate – has been proposed as central to the progression of OA.^[16]^

The synovitis scoring system defined by Kern et al was developed to standardize and make the evaluation of joint diseases more reproducible, thereby contributing to diagnostic accuracy. It provides a semiquantitative assessment of the severity and characteristics of inflammation in the synovial membrane.^[23]^ In a study by Kern et al, which evaluated synovitis across different patient groups, the synovitis scores ranged from 0 to 6 in patients with OA, from 1 to 7 in patients with psoriatic arthritis, and from 0 to 9 in those with rheumatoid arthritis (RA).^[19]^ Overall, the mean synovitis score in OA patients is typically around 2, indicating low-grade inflammation, whereas higher scores (5 and above) in patients with inflammatory rheumatic diseases reflect more severe inflammation.

In this study, high-grade synovitis was observed in patients with grade 4 OA the mean synovitis score was found to be 4.9, with a median score of 5. These elevated values are likely associated with the advanced disease stage of the patient group and are consistent with the upper score ranges reported for late-stage OA using the synovitis scoring system defined by Krenn et al Furthermore, in a study by Ene et al that evaluated the histomorphological findings of OA based on disease stages, localized inflammation and mild structural changes (such as fibrosis and mononuclear cell infiltration) were observed in early-stage OA, whereas widespread and chronic inflammation, along with prominent fibrosis and vascularization, were identified in late-stage OA.^[24]^ These findings suggest that advanced-stage OA may exhibit synovial changes resembling those seen in inflammatory rheumatic diseases.

Histologically, the synovium of OA patients exhibits features such as synovial fibroblast proliferation, increased vascularity, thickening of the synovial lining, and inflammatory cell infiltration – even before significant cartilage degeneration becomes evident.^[6]^ In this study, stromal edema and increased vascularity were frequently observed; however, the most prominent finding was fibrosis, which was present at moderate to severe levels in approximately 90% of patients. These findings are consistent with previous literature suggesting that inflammation predominates in early-stage OA, while fibrosis and structural remodeling become more pronounced in late-stage disease.^[8,14,21,25]^ Fibrotic remodeling of the synovial membrane contributes to joint stiffness and may be a more significant source of pain in late-stage OA than inflammation. This reinforces the idea that, in addition to inflammatory pathways, targeting fibrotic mechanisms may offer promising therapeutic opportunities.

The interaction between IFP and the synovial membrane adds further complexity to the pathophysiology of OA. These 2 structures are anatomically and functionally interconnected, acting as a single unit. The IFP secretes pro-inflammatory adipocytokines and exhibits increased vascularity and fibrosis in OA.^[11–13]^ IFP inflammation has been shown to be associated with pain and may contribute to both peripheral and central sensitization in knee OA.^[12]^ Preventing this sensitization – which is linked to chronic and persistent pain – could represent a novel strategy for altering disease progression.

MGC’s have been identified in various types of arthritis since their first description in rheumatoid synovium. However, their role in the pathogenesis of OA or RA remains largely unclear.^[16,26]^ In a study by Prieto-Potin et al, it was demonstrated that cells of the mononuclear phagocyte system are activated in both the synovium and subchondral bone in OA and RA. The number of MGCs was found to be similar in both diseases. While both Langhans-type and foreign body-type giant cells were observed in RA, Langhans-type cells were predominantly found in OA. It has been suggested that MGC contribute to catabolic activity and are regulated by the synovium.^[27]^ In tis study, MGC were observed in 14% of cases. Moreover, these cells were predominantly of the Langhans type and were mostly found in patients with high synovitis scores and severe inflammation. The presence of MGC’s was statistically associated with the severity of inflammation and the total synovitis score (*P* < .001). Despite growing knowledge about these mechanisms, therapies targeting inflammation or fibrosis in OA have yet been developed. Future research should focus on longitudinal studies that monitor changes in synovial tissue from early to advanced stages of the disease, as well as on the identification of molecular targets within the synovium and IFP. Standardized histopathological scoring systems, such as the one used in this study, may enable consistent evaluation of synovial pathology and improve diagnostic accuracy in both clinical and research settings.

### 4.1. Strengths and limitations of the study

One of the main strengths of this study is its focus on a relatively underexplored topic – the histopathological evaluation of synovial tissue obtained during total knee arthroplasty for primary OA. The study provides valuable insights into synovial alterations associated with advanced-stage OA. However, a significant limitation is the absence of a control group, as all participants were patients with grade 4 OA. The lack of a comparator group (e.g., healthy individuals or patients with early-stage OA) limits the generalizability of the findings across the full clinical spectrum of the disease. To address this limitation, relevant data from existing literature on early-stage OA were incorporated to contextualize the findings.^[15,18]^ Furthermore, the patient cohort in this study represents a broader range of individuals compared to previous studies, contributing meaningfully to the understanding of histomorphological features characteristic of advanced-stage OA.

### 4.2. Implications for clinical practice and research

The findings carry several potential clinical implications. Most notably, the presence of severe fibrosis in advanced-stage OA suggests that fibrotic processes may play a more significant role in pain generation than inflammation in this patient group. Given that fibrosis is a hallmark of advanced disease, targeting fibrosis-associated pathways could support the development of alternative treatment approaches for OA. The results also highlight the importance of implementing standardized histopathological criteria for synovitis assessment in OA. Future studies should prioritize the longitudinal evaluation of synovial tissue across various stages of OA to better understand the temporal dynamics of inflammation and fibrotic remodeling.

## 5. Conclusions

This study highlights the importance of histopathological analysis in improving the understanding of OA progression. The findings indicate that synovial fibrosis may represent a prominent feature of advanced-stage OA and could have implications for both pain mechanisms and disease management. Moreover, the presence of moderate synovial inflammation observed in this study further emphasizes the role of synovitis in OA pathogenesis. The use of synovitis scoring systems provides a practical and standardized approach to evaluating synovial tissue in both clinical and research settings. Identifying and targeting potential mechanisms driving fibrosis may support the development of new therapeutic strategies, particularly for patients with advanced OA.

Future research should prioritize both the investigation of fibrosis-related biological pathways as therapeutic targets and longitudinal studies aimed at elucidating the temporal dynamics of inflammation and fibrotic remodeling across different stages of OA.

## Acknowledgments

The author thanks Prof Dr Cem Zeki Esenyel (Orthopaedic Surgeon) for his support and guidance during the preparation of the manuscript. He has reviewed this statement and agreed to be acknowledged by name.

## Author contributions

**Conceptualization:** Ilkay Cinar.

**Data curation:** Ilkay Cinar.

**Formal analysis:** Ilkay Cinar.

**Writing – original draft:** Ilkay Cinar.

**Writing – review & editing:** Ilkay Cinar.
